# Coexistence of lymphoplasmacytic lymphoma and multiple myeloma: a case report and review of literature

**DOI:** 10.3389/fonc.2026.1789073

**Published:** 2026-06-26

**Authors:** Xiao-Qin Yang, Ting-Ting Liu, Hai-Yun Liu, Liang Li

**Affiliations:** 1 Department of Laboratory Medicine, Jiangxi Provincial People's Hospital / The First Affiliated Hospital of Nanchang Medical College, Nanchang, China; 2 Department of Hematology, Jiangxi Provincial People's Hospital / The First Affiliated Hospital of Nanchang Medical College, Nanchang, China; 3 Department of Laboratory Medicine, Jinxi County Hospital of Traditional Chinese Medicine, Fuzhou, China

**Keywords:** lymphoplasmacytic lymphoma, multiple myeloma, MYD88 L265P, Waldenström’s macroglobulinemia, zanubrutinib

## Abstract

Lymphoplasmacytic lymphoma (LPL) and multiple myeloma (MM) are distinct B−cell neoplasms whose synchronous occurrence is extremely rare. We report a case of a 68−year−old man presenting with fatigue and bone pain. Laboratory studies showed hyperglobulinemia (71.1 g/L), severe normocytic anemia (hemoglobin 44 g/L, MCV 79.5 fL), and thrombocytopenia. Serum immunofixation revealed dual IgG−kappa and IgM−kappa monoclonal proteins. Imaging showed diffuse osteolytic lesions in the skull and spine. Bone marrow flow cytometry identified two immunophenotypically distinct populations: CD19+/CD20+/CD5-/CD10- B−cells (34.1%) and CD138+/CD38+/cytoplasmic kappa+ plasma cells (4.5%). Next−generation sequencing on unsorted whole bone marrow detected MYD88 c.779T>C (p.Leu260Pro, canonical L265P) with a variant allele frequency (VAF) of 40.00% and CXCR4 c.1013C>G (p.Ser338*) nonsense mutation with VAF 15.53%, confirming LPL. Although the clonal plasma cell percentage was below the traditional 10% threshold, the presence of unequivocal osteolytic lesions and severe anemia (CRAB criteria) supported a diagnosis of active MM. The patient was treated with zanubrutinib, bortezomib, and dexamethasone, achieving a partial response. This case strongly suggests that LPL and MM can coexist, but definitive proof of biclonality would require sorted−cell molecular studies. We highlight the diagnostic challenges and the importance of integrating clinical, immunophenotypic, and molecular data.

## 1 Introduction

Lymphoplasmacytic lymphoma (LPL) is an indolent B−cell neoplasm characterized by bone marrow infiltration of small B−lymphocytes, plasmacytoid lymphocytes, and plasma cells. Over 90% of cases are associated with a serum IgM paraprotein, defining Waldenström’s macroglobulinemia (WM). The discovery of the MYD88 L265P mutation, present in over 90% of WM cases, has become a pivotal diagnostic and pathogenetic marker (1). Multiple myeloma (MM) is a malignant proliferation of clonal plasma cells, typically producing IgG or IgA paraproteins and associated with end−organ damage (hypercalcemia, renal insufficiency, anemia, bone lesions – the “CRAB” criteria) (2).

The simultaneous presentation of LPL and MM is exceedingly rare. A systematic literature review identified only three well−documented cases of synchronous LPL/WM and MM with molecular confirmation (3–5). An additional large retrospective study on IgM myeloma provides important differential diagnostic context (6). More recently, Itchaki et al. reported a case series of coexisting LPL and MM, further supporting the reality of this overlap syndrome (13). Here we report a patient with concurrent MYD88 L265P−mutant LPL and an IgG−kappa plasma cell neoplasm, discuss the differential diagnosis (particularly plasmacytic differentiation within LPL/WM versus true biclonal disease), and emphasize the limitations of current diagnostic approaches.

## 2 Case presentation

A 68−year−old Chinese male was transferred to the hematology department with progressive fatigue and generalized bone pain. He had no history of hypertension, diabetes, or hyperlipidemia. His past medical history included cerebral artery stenosis (managed conservatively without antiplatelet therapy) and a remote episode of shingles. He was not taking any regular medications prior to admission. Physical examination revealed pallor but no palpable lymphadenopathy or hepatosplenomegaly.

Initial laboratory tests showed severe normocytic anemia (hemoglobin 44 g/L, MCV 79.5 fL; reference 80–100 fL) and thrombocytopenia (platelets 81×10^9^/L). Total protein was 90.5 g/L, globulin 71.1 g/L. Renal function and calcium were normal. Serum protein electrophoresis (SPEP) showed an M−spike of 48.8 g/L (57.4% of total protein). Immunofixation confirmed two distinct M−proteins: IgG−kappa (predominant) and IgM−kappa. Quantitative immunoglobulins: IgG 60.65 g/L (markedly elevated), IgA 0.28 g/L (suppressed), IgM 2.02 g/L (normal). Serum free light chains (sFLC): kappa 33.96 mg/L, lambda 17.59 mg/L, kappa/lambda ratio 1.93 (elevated). Beta−2−microglobulin was 2.90 mg/L; lactate dehydrogenase was normal.

**Figure 1:**
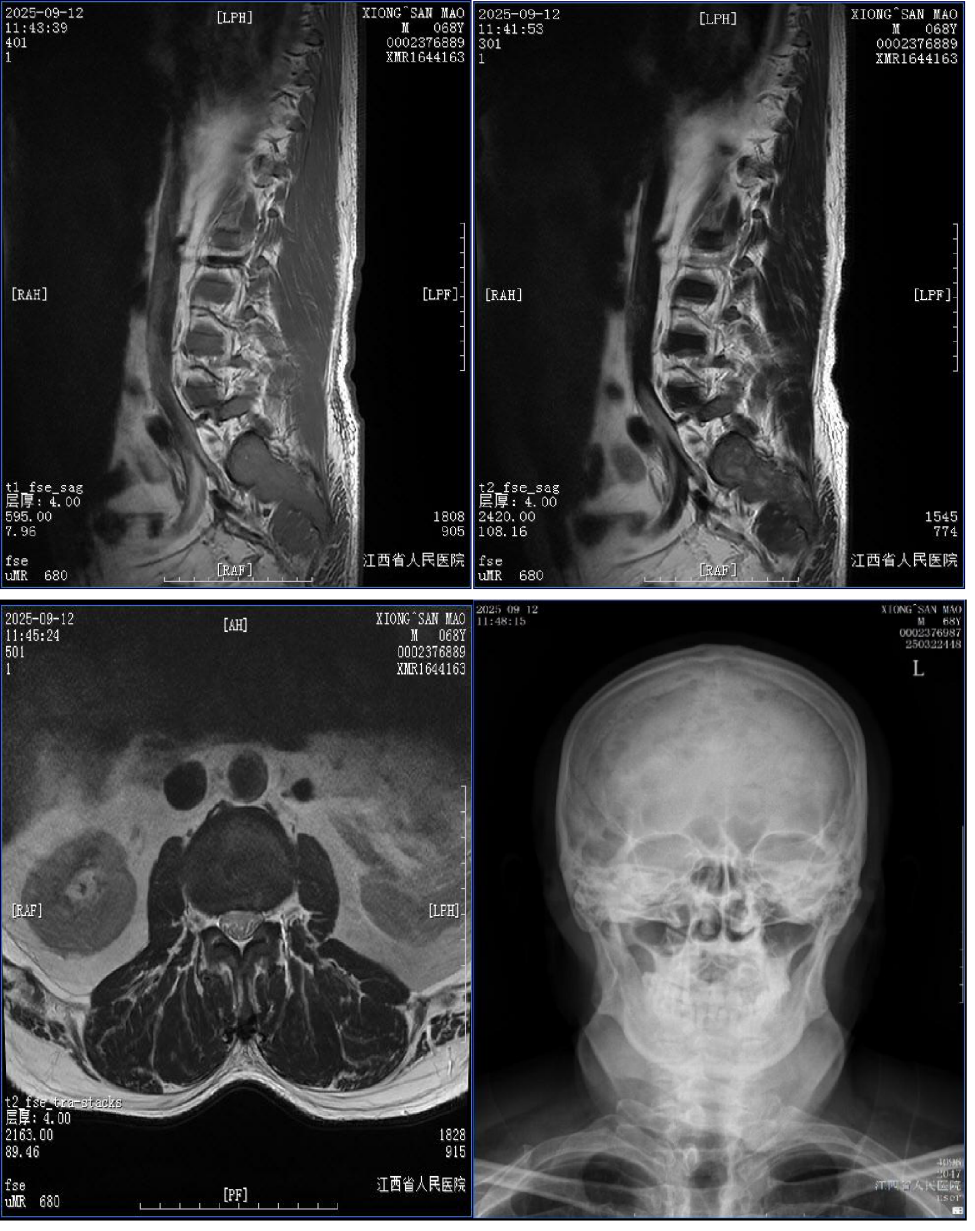
MRI of the lumbar spine showing diffuse heterogeneous signal intensity in the vertebral bodies on STIR sequence, consistent with myelomatous infiltration. X-Ray Scans: Round-like low-density shadows are seen in the diploë of the skull.

Whole−body MRI of the thoracolumbar spine showed diffuse heterogeneous signal on STIR sequences, suggestive of infiltrative disease. Skull X−ray revealed well−defined round lucencies (osteolytic lesions) in the diploë. CT of chest and abdomen showed no significant lymphadenopathy. No bone lesion biopsy was performed (**Figure 1**).

**Figure 2:**
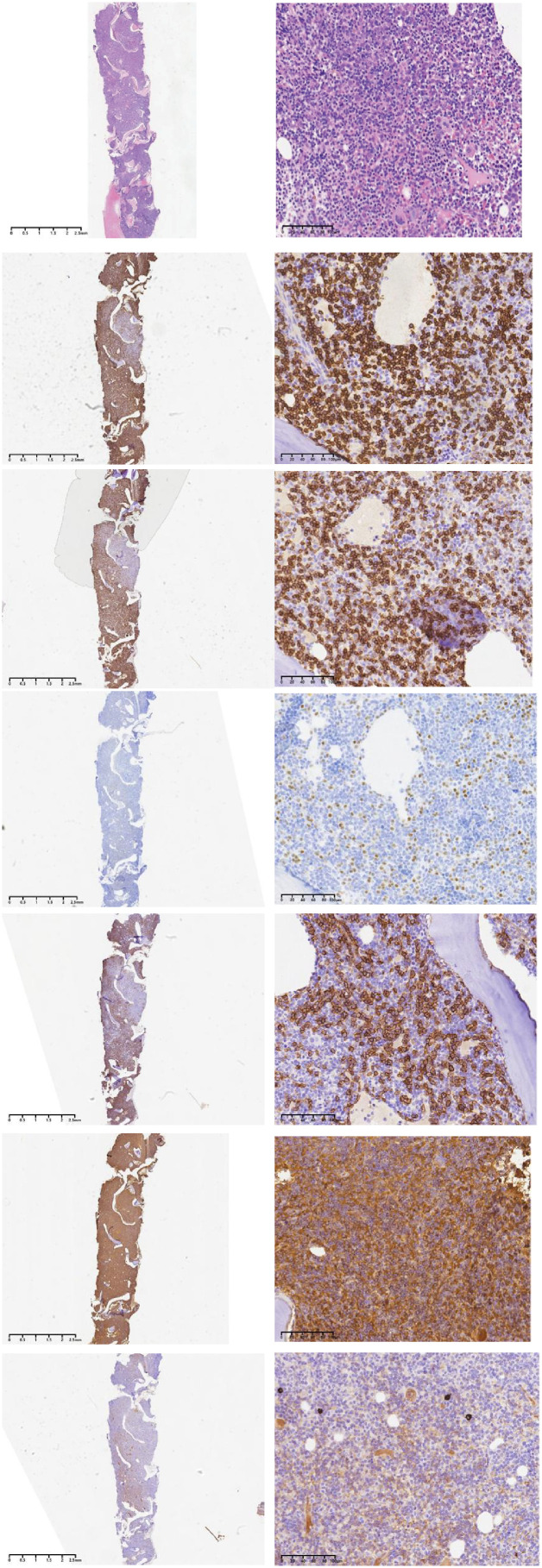
The panels show hematoxylin and eosin (H&E) staining and immunohistochemical staining for CD20, CD19, MUM1, CD138, κ light chain, and λ light chain. Each marker is presented at two magnifications: left column 1×10; right column 20×10.

**Figure 3:**
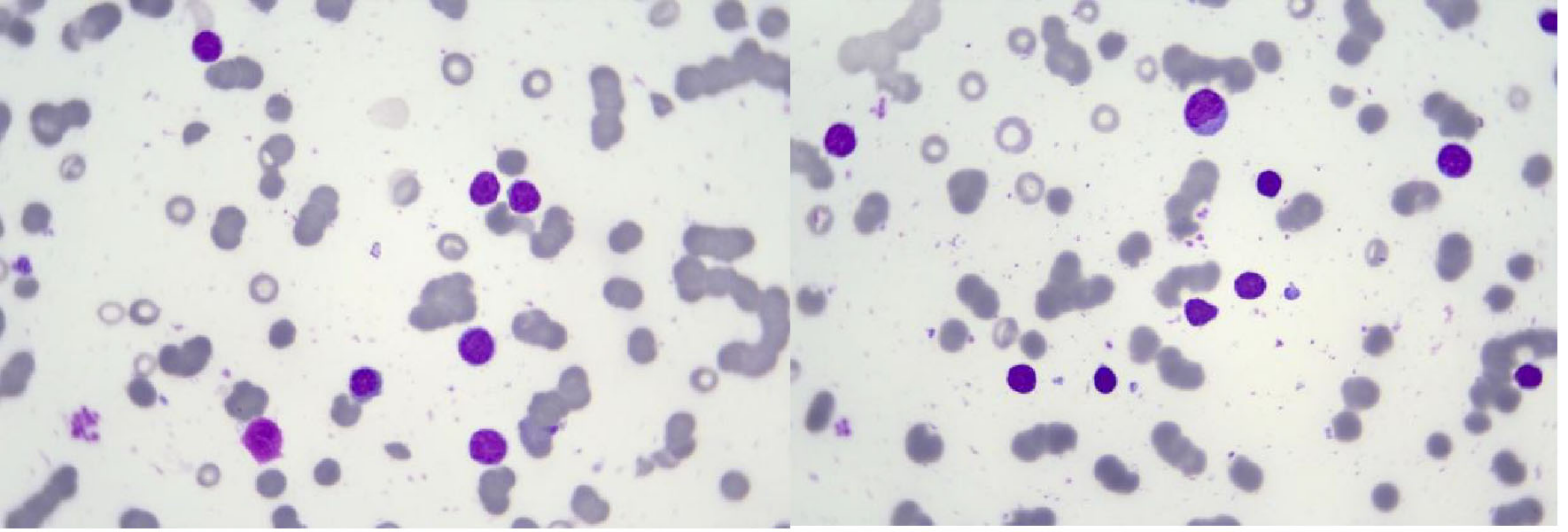
The bone marrow morphology was characterized by an infiltrate predominated by small lymphocytes (78%), with a minor component of plasmacytoid lymphocytes (5%).

Bone marrow aspiration and biopsy were performed. Morphology demonstrated a predominant infiltrate of small lymphocytes (78%) with occasional plasmacytoid lymphocytes (5%). Trephine biopsy showed diffuse infiltration by a low−grade B−cell lymphoma occupying approximately 70% of marrow cellularity. Immunohistochemistry: the neoplastic cells were positive for CD20, CD19, PAX5, CD79a, BCL2, and focally MUM1; negative for CD3, CD5, CD10, CD23, cyclin D1, BCL6, and λ light chain. CD138 highlighted scattered plasma cells, which showed κ light chain restriction. Ki−67 was 10% in tumor cells. EBER−ISH was negative (**Figures 2**, **3**).

**Figure 4:**
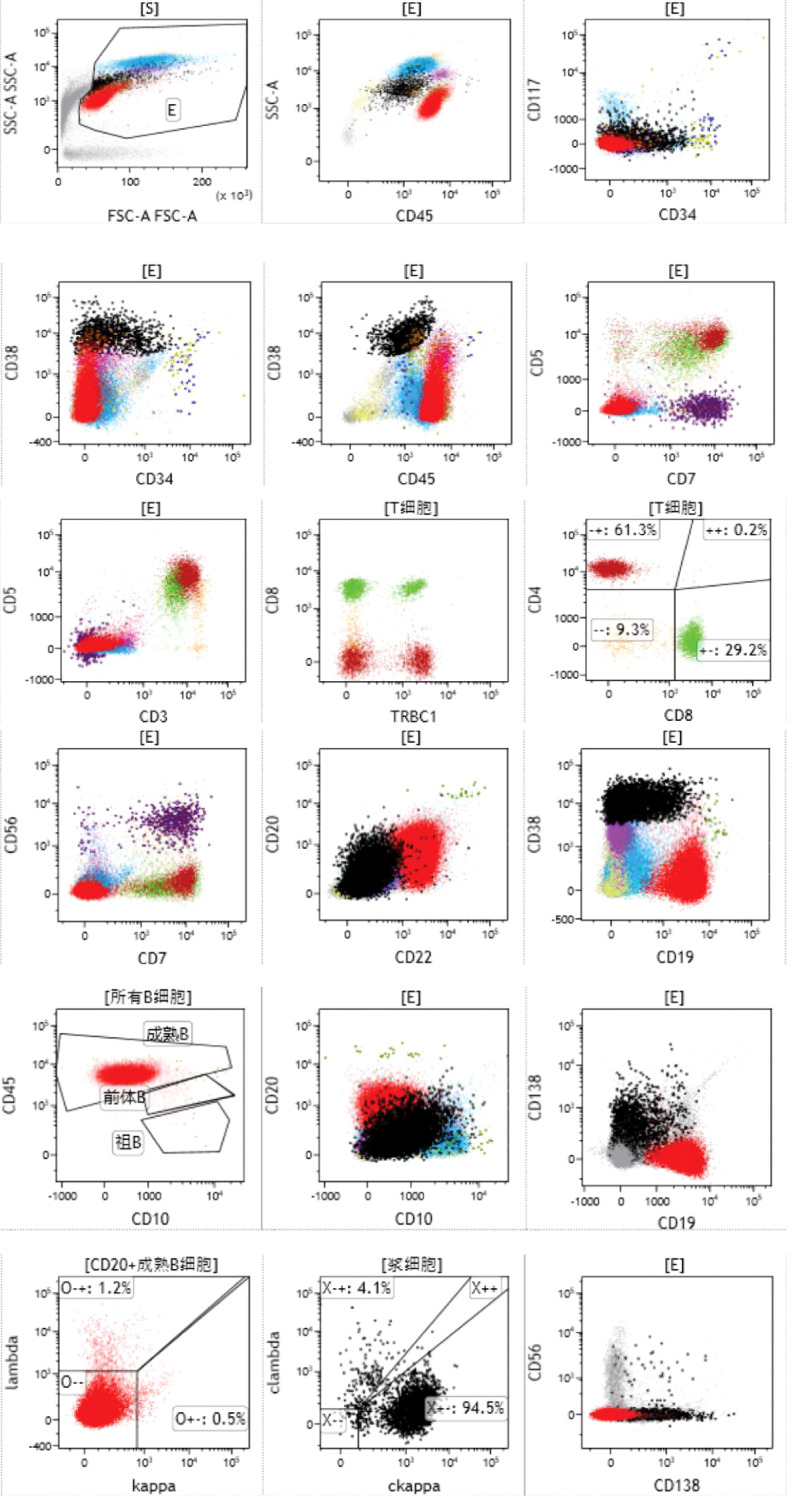
Flow cytometric plots. (A. red) Gating on the lymphocytic population (CD19+) showing expression of CD20 and lack of CD5/CD10. (B. black) Gatin on the plasma cell population (CD138+) showing restricted cytoplasmic kappa light chain expression.

Multiparameter flow cytometry (BD FACSCanto I) was performed on fresh bone marrow aspirate (**Figure 4**). A total of 50,000 events were acquired. The antibody panel included CD10, CD117, CD123, CD138, CD19 (two clones), CD20, CD22, CD3, CD33, CD34, CD36, CD38 (two clones), CD4, CD45 (four clones), CD5, CD56 (two clones), CD7, CD8, HLA−DR, TRBC1, cytoplasmic kappa (ckappa), cytoplasmic lambda (clambda), surface kappa, and surface lambda. Gating strategy: CD45 versus side scatter (SSC) for leukocytes; CD19+ for B−cells; CD138+ for plasma cells. Two aberrant populations were identified: Population A (LPL clone; 34.1% of nucleated cells): Small size, positive for HLA−DR, CD19, CD45; weakly positive for CD20 and CD22; negative for CD34, CD117, CD123, CD36, CD33, CD8, CD7, CD3, CD5, CD4, CD56, CD10, ckappa, clambda, surface kappa, surface lambda, CD38, TRBC1, and CD138. This phenotype is consistent with a CD5−/CD10− mature small B−cell neoplasm (LPL). Population B (plasma cell clone; 4.5% of nucleated cells, 100% of plasma cells): Positive for ckappa, CD38 (bright), CD138 (bright); weakly positive for CD45; negative for CD34, CD117, CD123, CD36, CD33, HLA−DR, CD8, CD7, CD3, CD5, CD4, CD56, CD19, CD20, CD22, CD10, clambda, surface kappa, surface lambda, and TRBC1. The CD45−dim, CD19−negative, CD56−negative, CD117−negative, CD38−bright, CD138−bright phenotype with cytoplasmic kappa restriction is characteristic of a clonal plasma cell neoplasm (MM phenotype). Normal T−cell, NK−cell, myeloid, and erythroid populations showed no significant aberrancies.

Molecular testing was performed on unsorted whole bone marrow aspirate using the Salus EVO next−generation sequencing platform with a 164−gene B−cell lymphoma panel (average depth 1621×, 200× coverage 99.81%). MYD88 c.779T>C (p.Leu260Pro), equivalent to the canonical L265P mutation, was detected with a variant allele frequency (VAF) of 40.00%. CXCR4 c.1013C>G (p.Ser338*), a nonsense mutation, was detected with a VAF of 15.53%. No other clinically actionable mutations were identified. Because testing was on unsorted whole marrow, the mutational status of the plasma cell clone could not be determined independently.

According to the International Myeloma Working Group (IMWG) criteria (7), the diagnosis of active MM requires ≥10% clonal plasma cells in marrow OR a biopsy−proven plasmacytoma plus myeloma−defining events (CRAB). In our patient, clonal plasma cells were only 4.5%, but there were unequivocal osteolytic lesions (skull lucencies) and severe anemia (CRAB). No plasmacytoma biopsy was obtained. While the MM diagnosis is plausible, we acknowledge that an atypical presentation of plasmacytic LPL/WM with osteolytic lesions cannot be entirely excluded. Therefore, we describe the case as concurrent LPL and a clonal plasma cell neoplasm highly suggestive of MM.

Given the CRAB criteria, immediate treatment was initiated. The patient received zanubrutinib (160 mg twice daily), bortezomib (1.3 mg/m² subcutaneously weekly), and dexamethasone (20 mg weekly). Lenalidomide was omitted due to baseline thrombocytopenia. Rituximab was deferred given the aggressive plasma cell component. After 4 cycles, the patient achieved a partial response: IgG decreased to 25.10 g/L, M−protein to 15.2 g/L, sFLC ratio normalized, hemoglobin increased to 112 g/L, platelets to 150×10^9^/L. Bone marrow flow cytometry showed reduction of abnormal B−cells to 5.5% and clonal plasma cells to 0.8% (very good partial response by IMWG criteria). The combination was well tolerated with no grade ≥3 adverse events. MRD monitoring by flow cytometry and NGS is planned after cycle 6. **Tables 1** and **2** summarize the key diagnostic and response data.

**Table 1:** Summary of key diagnostic findings.

Parameter	Result	Interpretation
Serum IFE	IgG-κ & IgM-κ	Dual M-proteins
IgG level	60.65 g/L	Markedly elevated
Bone marrow biopsy	70% B-cell infiltration	Low-grade B-cell lymphoma
Flow cytometry (B-cells)	34.1%, CD19+/CD20+/CD5-/CD10-	LPL immunophenotype
Flow cytometry (plasma cells)	4.5%, CD138+/CD38+/cκ+	MM immunophenotype
MYD88 L265P (c.779T>C)	Positive, VAF 40.00%	Molecular confirmation of LPL
CXCR4 S338* (c.1013C>G)	Positive, VAF 15.53%	Nonsense mutation in LPL
MRI spine	Diffuse infiltrative changes	Myelomatous bone disease

**Table 2:** Treatment response.

Parameter	Baseline	Follow-up	Reference range	Response assessment
Clinical status				
ECOG PS	2	1	0–1	Improved
Bone pain	Severe	Mild	Absent	Improved
Serum M-protein studies				
IgG (g/L)	60.65	25.10	7.51–15.60	Partial response (MM component)
IgM (g/L)	2.02	0.80	0.46–3.04	Normalized
M-protein (g/L)	48.8	15.2	0	Partial response (MM component)
M-protein (%)	57.4	20.1	0	Partial response (MM component)
Light chain studies				
sFLC κ/λ ratio	1.93	1.25	0.26–1.65	Normalized
sFLC κ (mg/L)	33.96	16.50	3.80–19.20	Normalized
sFLC λ (mg/L)	17.59	10.95	7.20–25.80	Normalized
Hematology				
Hemoglobin (g/L)	44	112	130–175	Hematological improvement
Platelets (×10^9^/L)	81	150	120–300	Normalized
WBC (×10^9^/L)	3.90	5.80	3.5–9.5	Normalized
Other biomarkers				
β2-microglobulin (mg/L)	2.90	1.80	1.0–3.0	Improved
LDH (U/L)	145	135	120–250	Normal
Creatinine (μmol/L)	78	75	57–111	Normal
Bone marrow assessment				
Abnormal B-cells (flow, %)	34.1	5.5	Not detected	VGPR (LPL compartment)
Clonal plasma cells (flow, %)	4.5	0.8	Not detected	VGPR (MM compartment)

## 3 Discussion

We present a case of a patient with synchronous MYD88 L265P−mutant LPL and a clonal IgG−kappa plasma cell neoplasm with osteolytic lesions and anemia. This case highlights diagnostic challenges, particularly the distinction between true biclonal disease (two independent neoplasms) versus plasmacytic differentiation within LPL/WM.

Diagnostic rigor and limitations: The presence of two distinct M−proteins (IgM and IgG) and two immunophenotypically separate populations by flow cytometry strongly favors the coexistence of two clones. However, definitive proof would require sorted−cell molecular studies (e.g., single−cell MYD88 genotyping or IGH clonality analysis on isolated CD138+ plasma cells). Because molecular testing was performed on unsorted whole marrow, we cannot state with certainty that the plasma cells are MYD88−wild type or that they represent a fully independent MM clone. Plasmacytic differentiation in WM/LPL can occasionally produce osteolytic lesions, although this is extremely rare. Therefore, our conclusion is tempered: the case provides strong evidence for coexistence, but not unequivocal proof. This limitation is now explicitly acknowledged.

Multiple myeloma diagnosis in the context of low plasma cell percentage: The IMWG 2014 criteria (7) require ≥10% clonal plasma cells for MM diagnosis in most situations, except when a plasmacytoma is biopsy−proven. Our patient had only 4.5% clonal plasma cells, but the skull X−ray showed typical osteolytic lesions (round lucencies) and MRI showed diffuse marrow abnormalities. No bone biopsy was performed, which is a limitation. Nevertheless, the combination of a clonal IgG−kappa plasma cell population, CRAB−defining severe anemia, and lytic bone lesions makes MM the most likely diagnosis, although an aggressive plasmacytic variant of LPL/WM cannot be ruled out.

Literature review: We performed a systematic PubMed search (keywords: “Waldenström macroglobulinemia AND multiple myeloma”, “lymphoplasmacytic lymphoma AND plasma cell myeloma”, “MYD88 AND biclonal gammopathy”) up to May 2026. Seven well−documented cases of synchronous or metachronous LPL/WM and MM were identified (**Table 3**). Among these, only three had MYD88 testing, all positive in the LPL component. Our case adds to this limited literature.

**Table 3:** Literature review of reported cases of synchronous LPL/WM and MM with *MYD88* confirmation.

Characteristic	Wang et al. (2012) (3)	Awada et al. (2019) (4)	Varettoni et al. (2013) (5)	Present case (2026)
Age/Sex	70/M	74/M	65/F	68/M
Paraproteins (WM/MM)	IgMλ/IgAλ	IgMκ/IgGκ	IgMκ/IgGκ	IgMκ/IgGκ
MYD88 status	Not tested	Positive in LPL	Positive in LPL	Positive in LPL (VAF 40%)
Bone marrow findings	LPL + 10% plasma cells	LPL + 30% plasma cells	LPL + 15% plasma cells	LPL (34.1%) + clonal plasma cells (4.5%)
Bone lesions	Yes	Yes	No	Yes (skull, spine)
Treatment	Chemotherapy	Ibrutinib + bortezomib/dex	R-CHOP	Zanubrutinib + bortezomib/dex
Outcome	NR	PR	CR	PR (MM), VGPR (LPL)

WM, Waldenström’s macroglobulinemia; MM, multiple myeloma; LPL, lymphoplasmacytic lymphoma; VAF, variant allele frequency; PR, partial response; CR, complete response; VGPR, very good partial response; R-CHOP, rituximab-cyclophosphamide-doxorubicin-vincristine-prednisone; NR, not reported.

Pathogenesis: The origin of such composite B−cell/plasma cell neoplasms is debated. LPL/WM is thought to arise from post−germinal center B−cells that have undergone somatic hypermutation but not isotype switching. MM typically arises from more differentiated post−switched plasma cells. The shared κ light chain restriction in our case raises the possibility of a common clonal origin followed by divergent evolution. The MYD88 L265P mutation activates NF−κB and promotes B−cell survival; additional genetic events (e.g., NRAS, KRAS, TP53, IRF4 rearrangements) could drive plasma cell differentiation and isotype switching from IgM to IgG (8). Conversely, two independent primary malignancies (collision tumors) are statistically unlikely but possible. Future studies using single−cell multi−omics are needed to resolve clonal relationships.

Therapeutic rationale: Our regimen (zanubrutinib + bortezomib + dexamethasone) was chosen based on NCCN guidelines and the WM consensus recommendations (12) for MYD88−mutant LPL (BTK inhibitor), and on MM guidelines (proteasome inhibitor). Lenalidomide was avoided due to thrombocytopenia (platelets 81×10^9^/L) and risk of myelosuppression. Rituximab was omitted initially to avoid potential IgM flare and to focus on rapid control of the IgG plasma cell clone; it could be added later if LPL dominates. The combination of zanubrutinib and bortezomib has been reported in small series with acceptable safety (9). We monitored for peripheral neuropathy and neutropenia; none occurred. MRD assessment will be performed by next−generation sequencing of IGH rearrangements.

Comparison with current classifications: According to the WHO 5th edition classification (10) and the International Consensus Classification (11), the immunophenotype of LPL (CD19+, CD20+, CD5−, CD10−, with MYD88 mutation) and MM (CD38+, CD138+, CD19−, with light chain restriction) are well defined. Our case fulfills both sets of criteria, supporting the diagnosis of two concurrent neoplasms.

## 4 Conclusion

This case strongly suggests the coexistence of MYD88 L265P−mutant LPL and an IgG−kappa plasma cell neoplasm with MM−defining features. However, definitive proof of biclonality requires sorted−cell molecular analysis. Clinicians should be aware of this rare overlap syndrome, especially when dual paraproteins or atypical bone lesions are present. A comprehensive diagnostic approach integrating morphology, flow cytometry, and molecular testing is essential. Future studies should employ single−cell techniques to clarify clonal relationships.

### 4.1 Methods (flow cytometry and molecular testing)

#### 4.1.1 Flow cytometry

Bone marrow aspirate (EDTA−anticoagulated) was processed within 24 hours. Flow cytometry was performed on a BD FACSCanto I instrument (BD Biosciences). A total of 50,000 events were acquired per tube. The antibody panel (30 markers) included: CD10, CD117, CD123, CD138, CD19 (two clones), CD20, CD22, CD3, CD33, CD34, CD36, CD38 (two clones), CD4, CD45 (four clones), CD5, CD56 (two clones), CD7, CD8, HLA−DR, TRBC1, ckappa, clambda, surface kappa, and surface lambda (all BD Biosciences). Gating strategy: leukocytes were identified by CD45 versus side scatter (SSC). B−cells were gated as CD19+ cells; plasma cells were gated as CD138+ cells. Fluorescence intensity was defined as bright (bri), dim (dim), or heterogeneous (het) relative to normal internal populations. Positivity thresholds: >80% for surface markers, >10% for cytoplasmic antigens; negativity defined as <20% for surface markers and <10% for cytoplasmic antigens.

#### 4.1.2 Molecular testing

DNA was extracted from 2 mL of EDTA−anticoagulated whole bone marrow aspirate (unsorted) using the QIAamp DNA Mini Kit (Qiagen). Targeted next−generation sequencing (NGS) was performed on the Salus EVO platform using a custom panel covering complete exonic regions of 164 B−cell lymphoma−associated genes, including MYD88 and CXCR4. Hybrid capture probes were used. Average sequencing depth was 1621× (range 900–2000×); 200× coverage of target regions was 99.81%; Q30 base quality was 90.35%. Variant calling used an in−house pipeline. Variant allele frequencies (VAFs) were calculated as the percentage of reads harboring the variant. Limit of detection for SNVs was ~1%–2% VAF. All variants were manually reviewed in IGV.

### 4.2 CARE checklist statement

This case report follows the CARE guidelines. The completed checklist is available as a supplementary file.

## Data Availability

The original contributions presented in the study are included in the article/**Supplementary Material**. Further inquiries can be directed to the corresponding author.
