# Supplementary material for: Coexistence of lymphoplasmacytic lymphoma and multiple myeloma: a case report and review of literature
Source: Front Oncol. 2026 Jun 26;16:1789073. doi: 10.3389/fonc.2026.1789073 (PMC13371161; doi:10.3389/fonc.2026.1789073)
Supplement: Supplementary file 1 [file Table1.docx]

**CARE Checklist: A Guide for Writing a Case Report**

**Manuscript Title:** Coexistence of Lymphoplasmacytic Lymphoma and Multiple Myeloma: A Case Report and Review of Literature
**Authors:** Xiao-Qin Yang, Ting-Ting Liu, Hai-Yun Liu, Liang Li
**Journal:** Frontiers in Oncology
**Date Prepared:** December 29, 2025

| **Item No.** | **Item Description** | **Location in Manuscript (Section/Page/Line)** | **Notes/Remarks** |
| --- | --- | --- | --- |
| **1** | **Title** – The words “case report” should be in the title. | Title | Yes |
| **2** | **Keywords** – 2–5 keywords to identify the report in literature searches. | Abstract – Keywords | Yes |
| **3** | **Abstract** – Provides a summary of the report including background, case presentation, and conclusions. | Abstract | Structured abstract with Background, Case Presentation, Conclusion. |
| **4** | **Introduction** – Briefly summarizes why this case is unique and its contribution to the literature. | Introduction | Provides background on LPL and MM, rarity of coexistence, and purpose of report. |
| **5** | **Patient Information** – Describes the patient’s demographic characteristics and main symptoms. | Case Presentation – First paragraph | Age, gender, presenting complaints (fatigue, bone pain), past medical history. |
| **6** | **Clinical Findings** – Describes relevant physical examination findings. | Case Presentation – Physical examination sentence | Pallor noted, no lymphadenopathy or hepatosplenomegaly. |
| **7** | **Timeline** – Relevant dates and times (e.g., history, assessments, interventions). | Implied in text (presentation, diagnostic workup, treatment initiation). Could be supplemented with a timeline figure. | Consider adding a supplementary timeline figure for clarity. |
| **8** | **Diagnostic Assessment** – Includes methods, results, and any diagnostic challenges. | Sections 2.1–2.3 (Laboratory, Imaging, Bone Marrow Studies) | Comprehensive workup detailed, including serology, imaging, flow cytometry, and molecular testing. |
| **9** | **Therapeutic Intervention** – Describes the type, dosage, and duration of treatment. | Section 2.5 (Treatment, Outcome, and Follow-up) – First paragraph | Regimen: Zanubrutinib + Bortezomib + Dexamethasone; supportive care specified. |
| **10** | **Follow-up and Outcomes** – Reports clinical outcomes and any adverse events. | Section 2.5 (Treatment, Outcome, and Follow-up) – Follow-up data in Table 2 | Clinical and laboratory improvements documented at 4 cycles. |
| **11** | **Discussion** – Provides interpretation and context, including comparisons with literature, strengths, and limitations. | Discussion | Pathogenesis hypotheses, clinical lessons, literature review included. |
| **12** | **Patient Perspective** – Includes the patient’s own views and experiences, if available. | Not explicitly included. | Optional item; patient consent for publication was obtained. |
| **13** | **Informed Consent** – Confirms that informed consent was obtained from the patient. | Ethics Statement | Stated: “Written informed consent was obtained from the patient for publication.” |
| **14** | **Ethics Statement** – Indicates whether ethical approval was obtained and adherence to ethical guidelines. | Ethics Statement – Added sentence: “The study was conducted in accordance with the principles of the Declaration of Helsinki.” | Explicit statement now included. |
| **15** | **Conflicts of Interest** – Discloses any conflicts of interest. | Conflict of Interest section | “The authors declare no competing interests.” |
| **16** | **Funding** – Describes sources of funding for the work. | Funding Information section | Grant information provided. |
| **17** | **Author Contributions** – Describes the contribution of each author. | Author Contributions section | Roles specified. |
| **18** | **Data Availability** – States how data supporting the report can be accessed. | Data Availability Statement | Updated to: “Data sharing is not applicable to this article as no datasets were generated during the study. Supporting clinical data are available from the corresponding author on reasonable request.” |
| **19** | **References** – Provides a complete list of references. | References | Yes. |
| **20** | **Figures and Tables** – All figures and tables are numbered, have titles/legends, and are cited in the text. | Figures 1–4, Tables 1–2; all referenced in text (e.g., Table 2 cited in Section 2.5) | Yes. |
